# Clade II Mpox Infections Among Cruise Ship Passengers and Crew Members — United States, 2024

**DOI:** 10.15585/mmwr.mm7422a1

**Published:** 2025-06-19

**Authors:** Nancy Ortiz, Luis R. Rodriguez, Mecca McPherson, Kristen Pringle, Agam K. Rao, Alexandra Tuttle, Christine M. Hughes, Rachel E. Kachur, Laura A. S. Quilter, Alida Gertz, Francisco Alvarado-Ramy, Clive Brown, Sundari Mase, Kara Tardivel

**Affiliations:** ^1^Division of Global Migration Health, National Center for Emerging and Zoonotic Infectious Diseases, CDC; ^2^Kennedy Krieger Institute, Baltimore, Maryland; ^3^Division of High-Consequence Pathogens and Pathology, National Center for Emerging and Zoonotic Infectious Diseases, CDC; ^4^Division of STD Prevention, National Center for HIV, Viral Hepatitis, STD, and TB Prevention, CDC.

**Summary**

**What is already known about this topic?**

During the global clade II mpox outbreak, cases have disproportionately affected gay, bisexual, and other men who have sex with men (MSM). Cruise ship travel–associated mpox infections have not been previously described.

**What is added by this report?**

During January 25–April 18, 2024, CDC was notified of eight mpox cases on four cruise ships: four among crew members and four among passengers. All cases occurred among MSM; five of eight patients had not been vaccinated against mpox.

**What are the implications for public health practice?**

Cruise lines should consider educating crew members on symptoms, risks, and preventive measures related to mpox and working with medical personnel to facilitate mpox vaccination for eligible crew members. Cruise passengers who are recommended to get the vaccine should receive mpox vaccine before travel. For cruise voyages marketed to gay and bisexual men, mpox-prevention messaging and education before and during voyages are recommended.

## Abstract

During the global clade II mpox outbreak, cases have disproportionately affected gay, bisexual, and other men who have sex with men (MSM). Cruise ship travel–associated mpox infections have not been previously described. During January 25–April 18, 2024, CDC was notified of eight mpox cases among cruise travelers on four ships: four among crew members and four among passengers. Seven cases were laboratory-confirmed as clade II *Monkeypox virus*. All exposure histories indicated male-to-male sexual contact. No patients were hospitalized, and none died. Crew members with mpox received their diagnoses on board and were isolated while infectious. Contacts were identified, monitored, and assessed for mpox postexposure prophylaxis (mpox vaccination). No crew members with mpox had been vaccinated against mpox. Passengers with mpox received their diagnoses after cruising on voyages marketed to gay and bisexual men, with symptom onset dates suggesting voyage exposures. For one cruise ship, two of the three reports of mpox among passengers were received after health departments were notified of potential cruise-associated exposures, and letters were sent to other passengers. Three of the four passengers with mpox had received 2 doses of JYNNEOS vaccine in 2022. Cruise lines should consider educating crew members on symptoms, risks, and preventive measures related to mpox and working with medical personnel to facilitate mpox vaccination as preexposure prophylaxis for eligible crew members. Cruise passengers who are eligible, predominantly MSM, should receive mpox vaccine before cruise travel. For cruise voyages marketed to gay and bisexual men, having mpox vaccine available on board would facilitate timely postexposure prophylaxis, if indicated; mpox prevention messaging and education before and during a voyage are also recommended.

## Introduction

In May 2022, a global outbreak of clade II mpox emerged, resulting in approximately 100,000 cases in 122 countries. In the United States, mpox case counts peaked in summer 2022 and decreased by early 2023 but continue to be reported (*1*). As of June 1, 2025, approximately 35,000 mpox infections had been reported in the United States, predominantly affecting gay, bisexual, and other men who have sex with men (MSM) (*2*). Mpox disease is caused by the orthopoxvirus *Monkeypox virus*, and is diagnosed using real-time polymerase chain reaction (PCR) testing. Transmission occurs through close contact (including sexual or intimate contact) with an infectious person or contact with contaminated materials such as clothing or bedding (*3*). The disease can begin with prodromal symptoms including fever, malaise, chills, headache, or lymphadenopathy, followed by a disseminated rash that may be located on hands, feet, chest, face, or mouth or near the genitals, including penis, testicles, labia, vagina, and anus.* Illness typically lasts 2–4 weeks and, for the majority of patients, treatment is supportive including pain management. Severity of illness depends on the health of the patient and the site of exposure. Mpox infections associated with cruise ship travel have not been previously described.

## Investigation and Results

During January 25–April 18, 2024, CDC was notified of eight mpox infections among cruise travelers on four ships.^†^ Four infections were among crew members, including one three-person cluster. Passenger infections occurred on two cruises marketed to gay and bisexual men. Seven of the eight cases were laboratory-confirmed as clade II *Monkeypox virus*, the strain circulating globally (*3*); the other case was clinically compatible and met epidemiologic criteria, and therefore was classified as suspected.^§^ The patients were men aged 30–49 years, most of whom had rash and fever; none were hospitalized, and none died (Table 1). This activity was reviewed by CDC, deemed not research, and was conducted consistent with applicable federal law and CDC policy.^¶^

**TABLE 1:** Reported characteristics of patients with mpox (N = 8) associated with four cruise ship voyages — United States, 2024

Characteristic	No.
**Sex**	
Male	8
**Age group, yrs**	
30–39	5
40–49	3
**Traveler type**	
Crew member	4
Passenger	4
**Clinical signs and symptoms **	
Rash	7
Lesion, location	
Genitals	4
Face	3
Extremities	3
Trunk	2
Fever	6
Lymphadenopathy	3
Cough	3
Myalgia	2
Tenesmus	2
Malaise	1
Sore throat	1
Pruritus ani	1
Headache	1
**Outcome**	
Hospitalization	0
Death	0
Received preexposure prophylaxis*	3
Crew member (n = 4)	0
Passenger (n = 4)	3

* 2 doses of JYNNEOS vaccine.

### Cruise Ship A

On January 25, 2024, cruise ship A reported three crew members (patients 1–3) with rash, fever, lymphadenopathy, and cough to CDC; onset dates spanned January 18–25. On January 31, PCR testing detected orthopoxvirus in all three crew members. CDC confirmed clade II *Monkeypox virus* for all specimens.

Cruise ship A medical personnel isolated patients and interviewed them to ascertain exposures, identify contacts, and categorize their exposure risk. MicrobeTrace (version 9.0; CDC) (*4*) was used to visualize the contact-tracing network (Figure). The network consisted of 19 persons: the index patient (node 1) reported sexual contact** with two partners (nodes 2 and 3), resulting in a three-patient cluster with 16 other contacts (nodes A–P). The second patient (node 2) reported an additional sexual partner (node N). Remaining contacts had nonintimate exposures: seven health care workers (nodes F–L), six of whom (all but node L) were exposed to all patients; four cabinmates (nodes B, C, M, and O); two coworkers (nodes D and E); one friend (node P); and one cabin steward (node A).

**FIGURE:**
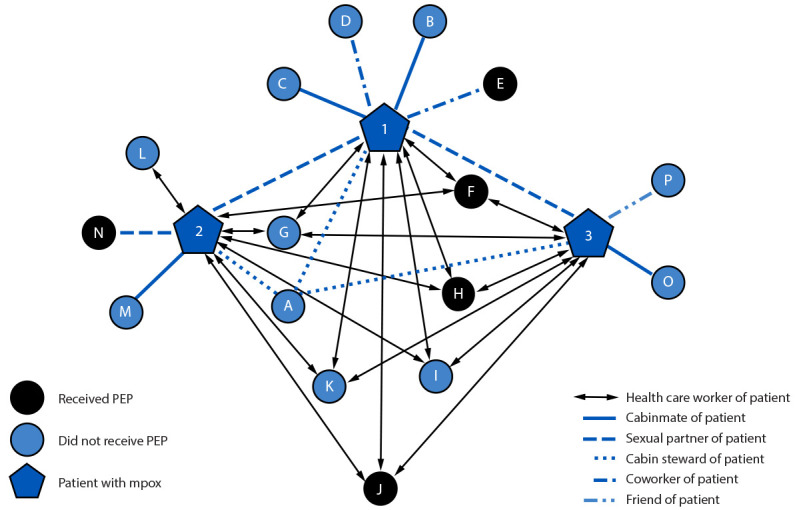
Contact-tracing network for three crew-member patients with mpox (nodes 1–3) and 16 contacts (nodes A–P) — cruise ship A, United States, January–February, 2024 **Abbreviation:** PEP = postexposure prophylaxis (JYNNEOS vaccine).

No crew-member patients with mpox or their contacts had received mpox vaccination as preexposure prophylaxis (PrEP) and none reported being immunocompromised. Cruise ship A procured and offered mpox vaccine as postexposure prophylaxis (PEP) to all identified contacts. Five of 16 received 2 doses of JYNNEOS vaccine: three health care workers (nodes F, H, and J) and one coworker (node E) received PEP during days 6–9 after exposure. One sexual partner (node N) received PEP 16 days postexposure (2 days beyond the recommended PEP window).^††^ Cruise ship A monitored contacts for mpox signs and symptoms until 21 days postexposure; no secondary cases were identified.

### Cruise Ship B

On February 7, 2024, a health department in the state of Washington notified CDC of a passenger with confirmed clade II mpox (patient 4) who developed symptoms of tenesmus and pruritus ani 2 days after voyaging on a cruise marketed to gay and bisexual men with approximately 5,000 passengers (cruise ship B). Patient 4 reported approximately 50 sexual partners during the voyage. Despite the absence of personally identifying information to fully trace contacts, CDC initiated public health interventions because of concerns about additional exposures. At CDC’s recommendation, the travel company distributed notification letters to all passengers and crew members regarding their *Monkeypox virus* exposure risk (Table 2). CDC obtained aggregate counts of the voyage’s passengers by U.S. state or country of residence and sent notifications to 58 U.S. state health jurisdictions via CDC’s Epidemic Information Exchange (Epi-X)^§§^ and to 68 countries’ International Health Regulations (IHR) National Focal Points. In addition, CDC notified the respective IHR National Focal Points for the three countries where cruise ship B had ported. On February 14, 2024, in response to the Epi-X notification, a California health department notified CDC of a second laboratory-confirmed clade II mpox case in a passenger on cruise ship B (patient 5) who experienced a genital rash on the day of disembarkation. Also on February 14, a Florida health department notified CDC of a suspected mpox case also in a cruise ship B passenger (patient 6), who experienced fever, myalgia, general pain, tenesmus, and a rash on the trunk, extremities, and perianal area 3 days after voyaging. Because patient 6’s specimen was not suitable for PCR testing, the case was categorized as suspected. Patients 5 and 6 reported having had 25 and four sexual partners during the voyage, respectively; neither provided personally identifying information for sexual partners. All three passenger patients associated with cruise ship B’s voyage had received 2 doses of JYNNEOS vaccine in 2022.

**TABLE 2:** Characteristics of patients with mpox and their contacts on four cruise ships and associated public health interventions, by voyage — United States, 2004

Cruise ship	Voyage type	No. of mpox cases		No. of contacts*				Public health intervention
		Crew member	Passenger	Received PrEP^†^	Identified	Sexual contact	Received PEP^†^	
A	Routine	3	0	0	16	1	5	Case isolation, contact tracing and monitoring, and mpox PEP
B	Marketed to gay and bisexual men	0	3	3	79	79	Unknown	Ship notification letters to passengers and crew members; CDC notifications to U.S. health departments and health authorities of affected countries
C	Marketed to gay and bisexual men	0	1	0	40	40	Unknown	None
D	Routine	1	0	0	8	0	0	Case isolation; contact tracing and monitoring

**Abbreviations**: PEP = postexposure prophylaxis; PrEP = preexposure prophylaxis.

* Contacts were enumerated while obtaining patients’ exposure histories or during contact tracing (cabinmates, cabin stewards, coworkers, friends, health care workers, and sexual partners).

^†^ 2 doses of JYNNEOS vaccine.

### Cruise Ship C

On March 26, 2024, a health department in Kentucky notified CDC of a confirmed clade II mpox case (patient 7) in a passenger who had traveled on a cruise marketed to gay and bisexual men with approximately 3,000 passengers (cruise ship C). His symptoms began 10 days after he concluded the voyage, and he reported having approximately 40 sexual partners on board. CDC received notification 31 days after the voyage (i.e., postincubation period). In addition, the notification occurred after the 14-day window for PEP administration. Hence, public health interventions were not initiated. The patient had no history of mpox vaccination.

### Cruise Ship D

On April 18, 2024, a California health department notified CDC of a confirmed clade II mpox case (patient 8) in a crew member on cruise ship D. On March 29, the patient went to the ship medical center with facial and extremity lesions and a history of sexual contact with a male partner before his March 19 ship embarkation; he reported no sexual contact while on board. CDC assisted cruise ship D with the case investigation, contact tracing, and PEP recommendations. Eight exposed crew-member contacts were identified; none reported history of mpox vaccination, and none was immunocompromised. No contacts met high or intermediate exposure risk criteria^¶¶^ for mpox; therefore, PEP was not recommended. Contacts were monitored for 21 days, and none developed mpox. Patient 8 reported that mpox vaccine for PrEP was not available in his home country.

## Discussion

This report highlights the potential for mpox transmission on cruise ships for both passengers and crew members. Prompt interventions that were conducted by cruise ship A might have prevented further transmission, including isolation of patients, contact tracing, and postexposure vaccination of unvaccinated contacts. None of the crew-member patients had previously completed the mpox vaccination series, despite being eligible to get the vaccine.***

Passenger patients were identified after voyaging on cruises marketed to gay and bisexual men. All developed symptoms on the day of disembarkation or after voyage completion, making it more likely that they were exposed, rather than sources of exposure, on the voyage; the index cases for these two voyages remained undetected. All identified passenger patients reported multiple sexual partners on board, suggesting widespread exposures and possibly additional undetected cases. Despite the lack of availability of personally identifying information to fully trace potential contacts, informational notifications identified two passenger patients.

Most of the passenger patients had received 2 doses of JYNNEOS vaccine in 2022. The occurrence of mpox infection in fully vaccinated persons is consistent with prior reports of infections in previously vaccinated persons, associated with multiple potential exposures (*5*). Although some persons might still become infected with mpox after completing the JYNNEOS vaccination series, mpox vaccination can help prevent illness, decrease disease severity, and prevent hospitalization and death (*5*–*7*).

Because the effectiveness of mpox vaccine for PEP is unclear and the challenge of identifying eligible persons within 4 days of exposure (when PEP is most effective), mpox vaccine as PrEP is preferred. Cruise lines should consider educating crew members on symptoms, risks, and preventive measures related to mpox and working with medical personnel to facilitate mpox vaccination as preexposure prophylaxis for eligible crew members, to help protect them from infection and reduce risk for potential onboard exposures. Cruise passengers who are eligible should receive mpox vaccine before cruise travel, with the second dose administered at least 2 weeks before travel to optimize effectiveness (*8*). Persons receiving mpox vaccine should be informed that infections might occur despite vaccination but could be less severe. For cruise voyages marketed to gay and bisexual men, having mpox vaccine available on board would facilitate timely PEP, if indicated; disseminating mpox prevention messaging and education^†††^ to passengers and crew members before and during a voyage are also recommended.

## Footnotes

*
Clinical Features of Mpox | Mpox | CDC

† Federal regulations (42 CFR 71.21) require the master of a ship sailing from a non-U.S. port and destined for a U.S. port to immediately report to CDC any death or certain illnesses among the ship's passengers or crew members, including travelers who have disembarked or were removed from the ship due to illness or death (Reporting Death or Illness on Ships | Port Health | CDC). In addition, CDC receives notifications from U.S. health departments of communicable disease of public health concern in recent travelers.

§
Mpox Case Definitions | Mpox | CDC

¶ 45 C.F.R. part 46. 102(I)(2), 21 C.F.R. part 56; 42 U.S.C. Sect. 241(d); 5 U.S.C. Sect. 552a; 44 U.S.C. Sect. 3501 et seq.

** Because mpox lesions occur on parts of the body not covered by barrier methods, information on known protective barriers, such as condoms, was not collected for any of the identified patients. Safer Sex, Social Gatherings, and Mpox | Mpox | CDC

††
Interim Clinical Considerations for Use of Vaccine for Mpox Prevention in the United States | Mpox | CDC

§§
Epi-X: The Epidemic Information Exchange

¶¶
Mpox Monitoring and Risk Assessment for People Exposed in the Community | Mpox | CDC

***
Mpox Vaccination | Mpox | CDC

†††
Preventing Mpox | Mpox | CDC
